# Memantine treatment for neuropsychiatric symptoms in a patient with probable idiopathic normal pressure hydrocephalus: a case report

**DOI:** 10.1186/1752-1947-7-94

**Published:** 2013-04-05

**Authors:** Masahiko Takaya

**Affiliations:** 1 Department of Neuropsychiatry, Kinki University School of Medicine, 377-2, Ohnohigashi, Osakasayama-shi, Osaka, 589-8511, Japan; 2 Department of Psychiatry, Osaka General Medical Center, 3-1-56, Bandaihigashi, Sumiyoshi-ku, Osaka, 558-8558, Japan

**Keywords:** Memantine, Neuropsychiatric symptoms, Idiopathic normal pressure hydrocephalus

## Abstract

**Introduction:**

Patients with idiopathic normal pressure hydrocephalus often show neuropsychiatric symptoms besides the triad of ‘classic’ symptoms. Memantine has been reported to have positive effects on the neuropsychiatric symptoms of patients with Alzheimer’s disease and patients with dementia with Lewy bodies. We administered memantine to a Japanese patient with probable idiopathic normal pressure hydrocephalus, hoping that this treatment would have positive effects on the neuropsychiatric symptoms of his idiopathic normal pressure hydrocephalus.

**Case presentation:**

An 80-year-old right-handed Japanese man was diagnosed as having probable idiopathic normal pressure hydrocephalus and showed neuropsychiatric symptoms as well as the triad of classic symptoms of idiopathic normal pressure hydrocephalus. We treated our patient with memantine by increasing, decreasing, and then again increasing the dose of memantine. We evaluated his neuropsychiatric symptoms using the Neuropsychiatric Inventory at baseline, after the dose was increased to 20mg/day, after the dose was decreased to 5mg/day, and after the dose was increased again to 15mg/day. We simultaneously evaluated the triad of symptoms and conducted neuropsychological tests. In addition, we evaluated the psychological distress of our patient’s caregiver using the Zarit Caregiver Burden Interview.

**Conclusions:**

Memantine had positive effects on the neuropsychiatric symptoms of our patient with idiopathic normal pressure hydrocephalus. Although none of his triad of classic symptoms, including cognitive abilities, improved, the psychological distress of our patient’s caregiver improved.

## Introduction

Normal pressure hydrocephalus (NPH) is characterized by a progressive neurological syndrome accompanied by a triad of symptoms: gait disturbances, cognitive impairment, and urinary dysfunction [1]. NPH is diagnosed as idiopathic NPH (iNPH) if a patient has no causative antecedent disease. Diagnosis and treatment of patients with iNPH has gained popularity, and shunt surgery is believed to be a very effective treatment for such patients [2]. However, there is no evidence to indicate whether shunt surgery is effective in the management of NPH [3]. Moreover, patients with iNPH or their caregivers do not necessarily desire surgical treatment or shunt surgery. In such cases, an attending psychiatrist often initiates psychotropic agents when a patient shows neuropsychiatric symptoms.

In addition to the triad of symptoms, neuropsychiatric symptoms are observed in patients with NPH [4]. The neuropsychiatric characteristics of patients with iNPH have been evaluated using the Neuropsychiatric Inventory (NPI) [5] and compared with those of patients of Alzheimer’s disease (AD) [6].

Various neuropsychiatric symptoms such as delusions, agitation, anxiety, and apathy are usually observed in patients with iNPH [6]. Memantine, an *N*-methyl-d-aspartate receptor antagonist, has significant effects on behavioral and psychological symptoms of dementia in patients with AD [7]. Because dementia with Lewy bodies (DLB) has some pathological and biochemical similarities to AD, pharmacological intervention with memantine might provide similar benefits for patients with DLB [8]. We hoped that memantine would therefore have similar effects on a patient with iNPH.

## Case presentation

We treated a case of probable iNPH from 1 December 2011 to 30 April 2012, according to the criteria of Relkin *et al*. [9].

**Figure 1:**
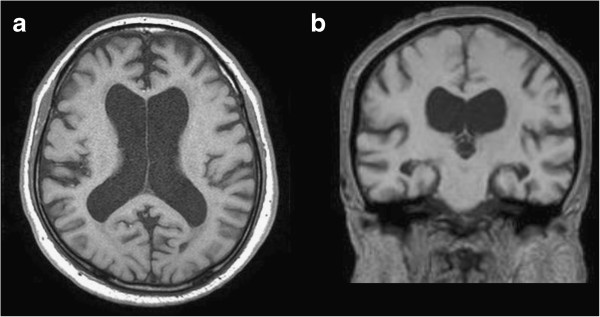
**Magnetic resonance imaging scans of our patient. **(**a**) Axial image showing lateral ventricular enlargement. (**b**) Coronal image showing narrowed subarachnoid spaces at the high convexity without severe cortical atrophy.

Our patient was an 80-year-old right-handed Japanese man. He complained of apathy, and took medication for hypertension. He had complained about amnesia and consulted a physician at a clinic four years ago, which he had regularly attended for treatment of hypertension three years previously. However, his activities of daily living were not remarkably impaired; therefore, his cognitive abilities were not evaluated by the physician. His amnestic complaints continued, and his family observed that his attention and memory had gradually become impaired and that his gait had gradually become unstable. His family persuaded him to consult a psychiatrist. Subsequently, he presented to our department, accompanied by his son and daughter-in-law eight months ago (that is, four years after he had first begun to complain about daily amnesia). He was diagnosed as having amnesia because he often could not recall names of his acquaintances. His gait disturbance complaint was based on the fact that he felt unstable, particularly going up and down stairs. We observed that he was apathetic, his gait was slightly ataxic, and he walked in a mildly wide-based manner. The results of brain magnetic resonance imaging scans showed lateral ventricular enlargement on an axial image, and narrowed subarachnoid spaces at the high convexity without severe cortical atrophy on a coronal image (Figure 1). The results of 99mTc-ethyl cysteinate dimer single photon emission computed tomography (SPECT) indicated nothing remarkable. His cerebrospinal fluid opening pressure was 15.5cmH_2_O, which was within the normal range (70 to 245mmH_2_O) [2]. Amnesia and inattention were observed at baseline neuropsychological tests, without disorientation to time and place (Table 1).

**Table 1:** Neuropsychological evaluation results

**Neuropsychological tests**	**Baseline score**	**Score on memantine (20mg/day)**
MMSE (scored out of 30)	20	18
ADAS (scored out of 70)	16.7	22.3
FAB (scored out of 18)	12	7
WMS-R: digit span (scored out of 24)	6	9
WMS-R: logical memory I: story immediate recall (scored out of 50)	0	0
WMS-R: logical memory II: story delayed recall (scored out of 50)	0	0
ROCFT (scored out of 36): Copy	34	30
ROCFT: Delayed recall	0	0
KBD: estimated intelligence quotient	37.5	45

ADAS, Alzheimer’s Disease Assessment Scale; FAB, Frontal Assessment Battery; KBD, Kohs block design test score; MMSE, Mini-Mental State Examination; ROCFT, Rey-Osterrieth Complex Figure Test; WMS-R, Wechsler Memory Scale - Revised.

We assessed our patient’s cognitive abilities using the Mini-Mental State Examination (MMSE), Alzheimer’s Disease Assessment Scale (ADAS), Frontal Assessment Battery (FAB), Wechsler Memory Scale - Revised (WMS-R), Rey-Osterrieth Complex Figure Test (ROCFT), and Kohs block design test (KBD). For the ADAS, higher scores indicate more severe cognitive impairment, while in the other tests lower scores indicate more severe cognitive impairment. In our patient’s case, the immediate recall and delayed recall scores on the MMSE were 3 out of 3 and 0 out of 3, respectively, indicating that his recent memory was impaired. His copy score on the ROCFT showed that his visuospatial ability was only slightly impaired. However, his delayed recall score on the ROCFT indicated that his recent memory was severely damaged. His digit span and story immediate recall scores revealed that his attention/concentration was severely impaired (Table 1).

His gait was unstable but independent. According to his caregiver, pollakiuria existed but urinary incontinence only occurred approximately once per month. Considering our patient’s clinical history, brain imaging data, and physical findings, we reached a diagnosis of probable iNPH, according to the criteria of Relkin *et al*. [9].

We evaluated the triad of symptoms using the iNPH Grading Scale (iNPHGS) [10]. The iNPHGS, which is a reliable and validated tool for assessing the triad of symptoms in these patients, has been used in many studies previously [6,11]. The score for each item ranges from 0 to 4 (higher scores indicate more severe symptoms). A zero score for an item indicates ‘normal’. On the basis of our patient’s clinical symptoms, we evaluated that the iNPHGS scores for cognitive impairment, gait disturbance, and urinary disturbance were 2, 2, and 1, respectively. Agitation, anxiety, apathy, and irritability were observed at baseline.

The NPI, which is a comprehensive tool for assessing various behavioral and psychiatric abnormalities in patients with organic mental disorders, such as dementia, has been used in many studies [12]. As in a previous study [5], we evaluated the neuropsychiatric characteristics of patients with iNPH using the NPI, in which the frequency score of each neuropsychiatric symptom ranges 0 to 4 (higher scores indicate more frequent symptoms), and the severity for each symptom ranges 0 to 3 (higher scores indicate more severe symptoms). Our patient’s scores for single NPI subitems are reported in Table 2.

**Table 2:** Neuropsychiatric Inventory scores for our patient

**Symptom**	**Memantine dose**							
	**0mg**		**20mg**		**5mg**		**15mg**	
	**F**	**S**	**F**	**S**	**F**	**S**	**F**	**S**
Delusion	0	0	0	0	0	0	0	0
Hallucination	0	0	0	0	0	0	0	0
Agitation	3	1	0	0	0	0	0	0
Depression	0	0	0	0	0	0	0	0
Anxiety	2	3	2	2	4	3	1	2
Euphoria	0	0	0	0	0	0	0	0
Apathy	4	2	2	1	4	2	1	2
Disinhibition	0	0	0	0	0	0	0	0
Irritability	4	2	0	0	0	0	0	0
Aberrant motor activity	0	0	0	0	0	0	0	0

Frequency (F) scores of 0, 1, 2, 3, and 4 indicate ‘not observed’, ‘observed occasionally, less than once per week’, ‘observed often, about once per week’, ‘observed frequently, several times per week but less than every day’, and ‘observed very frequently, once or more per day or continuously’, respectively. Severity (S) scores of 0, 1, 2, and 3 indicate ‘not observed’, ‘mild’, ‘moderate’, and ‘severe’, respectively. A dose of 0mg indicates baseline.

The Zarit Caregiver Burden Interview (ZBI) [12,13] was conducted to evaluate the psychological distress of our patient’s caregiver. The ZBI, which is a standardized, validated, and reliable tool for assessing the burden on caregivers of patients with dementia, has been widely used in many studies. It consists of a 22-item self-rating inventory to assess caregiver burden. The score of each item ranges from 0 to 4, and the total score ranges from 0 to 88. Higher scores indicate a more severe burden. In our patient’s case, the total score on ZBI was 67 out of 88, suggesting that our patient’s caregiver was exhausted.

After written informed consent, we started memantine treatment (5mg/day) and increased the dose gradually to 20mg/day. Our patient’s neuropsychological test scores did not improve one month after the dose reached 20mg/day (Table 1), whereas his NPI scores improved (Table 2). At this point, the iNPHGS scores were the same as those at baseline. However, somnolence was clearly observed one month after we increased the memantine dose to 20mg/day. Therefore, we gradually decreased the dose, and the somnolence disappeared completely when the dose was 5mg/day but our patient’s anxiety and apathy worsened. We increased the dose again, but somnolence was not observed even when the dose was 15mg/day, while anxiety and apathy decreased again. Agitation and irritability were persistently absent after initiation of therapy (Table 2). The ZBI was conducted again with the help of our patient’s caregiver, and the score was 44 out of 88. Compared to the baseline score, the psychological distress of our patient’s caregiver had apparently improved. To avoid side effects such as somnolence, we decided not to increase the dose to >15mg/day. Moreover, the iNPHGS scores were the same as those at baseline.

## Discussion

Memantine had positive effects on the psychiatric symptoms of agitation, anxiety, apathy, and irritability of our patient with iNPH. Moreover, the psychological distress of our patient’s caregiver seemed to improve, as assessed by the ZBI, and was accompanied by improved neuropsychiatric symptoms in our patient. This observation supports the hypothesis that administering memantine to patients with iNPH without surgical treatment could have significant positive effects on both patients and their caregivers. However, no effect of memantine could be detected on the triad of iNPH symptoms. Moreover, our patient’s cognitive status did not improve. Although our patient’s attention and estimated intelligence quotient seemed to improve as indicated by the WMS-R: digit span and KBD results, respectively, his scores on the MMSE, ADAS, FAB and ROCFT: copy were worse (Table 1). These results may have been within the range of test-retest error, or may be related to sedation linked to glutamate antagonist use.

We found no previous study in the literature that has reported the effects of memantine on neuropsychiatric symptoms in patients with iNPH. However, an effect of memantine on the neuropsychiatric symptoms of other dementias, such as AD [7] and DLB [8] has been reported. In particular, memantine has shown positive effects on agitation/aggression in patients with AD and DLB compared with those in a placebo group [8,14]. Memantine does not have beneficial effects on anxiety in patients with AD, but has positive effects on apathy [14]. In contrast, memantine has positive effects on anxiety in patients with DLB, but has opposite effects on apathy [8].

One strength of our present findings is represented by the observation of symptoms during the administration of different memantine dosages. This lends support to this being a true pharmacological effect, and suggests that the effects of memantine were not due to chance. Our patient took memantine after dinner every day during this trial. We decided that we might not be able to completely eliminate chance factor of somnolence.

We carefully treated only one patient with memantine, which resulted in improved neuropsychiatric symptoms, but no improvement in the triad of symptoms. The observed finding should be replicated to support the existence of a true therapeutic effect, which should be confirmed in a clinical trial.

## Conclusions

Memantine had positive effects on the psychiatric symptoms of a patient with probable iNPH, accompanied by improved psychological distress of our patient’s caregiver. However, no effect of memantine could be detected on the triad of iNPH symptoms or cognitive status.

## Consent

Written informed consent was obtained both from the patient and from his son for publication of this case report and any accompanying images. A copy of the written consent is available for review by the Editor-in-Chief of this journal.

## Abbreviations

AD: Alzheimer’s disease; DLB: Dementia with Lewy bodies; iNPH: Idiopathic NPH; iNPHGS: iNPH grading scale; NPH: Normal pressure hydrocephalus; NPI: Neuropsychiatric Inventory; ZBI: Zarit Caregiver Burden Interview

## Competing interests

The authors declare that they have no competing interests.
